# Preparation and Performance of Carbon-Based Ce-Mn Catalysts for Efficient Degradation of Acetone at Low Temperatures

**DOI:** 10.3390/ijerph192416879

**Published:** 2022-12-15

**Authors:** Tong Li, Zhibo Wang, Yue Shi, Xiaolong Yao

**Affiliations:** 1 School of Ecology and Environment, Beijing Technology and Business University, Beijing 100048, China; 2 SINOPEC (Beijing) Research Institute of Chemical Industry Co., Ltd., Beijing 100013, China; 3 State Environmental Protection Key Laboratory of Food Chain Pollution Control, Beijing Technology and Business University, Beijing 100048, China

**Keywords:** acetone, Ce_0.8_-Mn/AC, efficient degradation, low temperatures

## Abstract

Based on the porous carbon material from citric acid residue, catalysts of different Ce-Mn ratios were prepared with incipient-wetness impregnation (IWI) to delve into their acetone-degrading performance and relevant mechanisms. When the Ce-Mn molar ratio is 0.8, the prepared catalyst Ce_0.8_-Mn/AC shows abundant and uniformly dispersed Mn and Ce particles on the surface. The content of Mn and Ce on the Ce_0.8_-Mn/AC surface reaches 5.64% and 0.75%, respectively. At the acetone concentration of 238 mg/m^3^ (100 ppm), the laws of acetone degradation in different catalysts at different catalyzing temperatures and with various oxygen concentrations were studied, and we found that the rate of acetone degradation by Ce_0.8_-Mn/AC can exceed 90% at 250 °C. Cerium oxide and manganese oxide are synergistic in the catalytic degradation of acetone. Adding cerium to manganese-based catalysts can increase the oxygen migration rate in the catalysts and thus raise the reduction rate of lattice oxygen in manganese oxide. The results offer new ideas and approaches for the efficient and comprehensive utilization of bio-fermentation by-products, and for the development of cheap and high degradation performance catalysts for acetone.

## 1. Introduction

China is the world’s largest producer and exporter of citric acid, whose yield accounts for about 53% of the world’s total production [1]. In citric acid processing, bio-fermentation is the primary production process. The process produces both citric acid intermediates and many volatile organic by-products, among which acetone is one of the most considerable amounts [2,3]. Acetone can involve photochemical reactions [4,5], and can be oxidated to organic acids (such as formic acid and acetic acid) to promote the formation of secondary organic aerosols (SOA) [6,7]. Meanwhile, acetone is chronically toxic to human body, and can lead to chronic neurotoxicity and impair renal and liver functions [8,9]. Therefore, seeking the appropriate purification technology for acetone from citric acid production has become a significant issue in this research field.

Catalytic oxidation is one of standard methods to purify VOCs (volatile organic compounds), especially for the VOCs with low concentrations and no recycling value [10,11]. Catalytic performance of catalysts depends on the properties of active components in the catalyst. According to relevant degradation mechanisms of Mars–van Krevelen (MVK) [12,13], Langmuir–Hinshelwood (L-H) [13,14], and Eley–Ridea (E-R) [15], particles of the active components coat the supporting carrier surface in the preparation process. In the process of degradation, acetone molecules are converted into inorganic compounds through the interaction with lattice oxygen and adsorption oxygen on the surface of the catalyst carrier [16]. In the meantime, after the metal oxide releases lattice oxygen, oxygen vacancies will be formed on the catalyst surface [17]. These reduction centers are regenerated through the compensation of oxygen or oxygen atoms in the carrier gas. Therefore, the type and dispersibility of active components on the carrier surface and their ability to transfer electrons are the main influencing factors of acetone degradation [18].

Some researches indicated that the reduction catalysts carrying precious metals (gold, platinum, etc.) show good acetone-catalyzing performance and support acetone degradation at low temperatures [19,20,21]. However, they are usually expensive to prepare and prone to poisoning [22]. Therefore, catalysts carrying cheaper metallic oxides (ZnO, Mn_2_O_3_, CeO_2_, etc.) are more competitive in the purification of industrial waste gas [23,24,25,26]. Studies indicate that Cu can improve its oxygen storage capacity by reducing the activation energy of Ce^4+^ reduction during the catalytic oxidation of acetone with Cu_0.13_Ce_0.87_O_y_ [27]. The perovskite-type La_0.6_Pb_0.2_Ca_0.2_MnO_3_ catalyst has a large amount of manganese ions with different valances and can provide many oxygen vacancies to degrade over 95% acetone at 300 °C [28]. These results indicate that using Ce and Mn oxides as active components is possible to effectively degrade acetone.

On the other hand, appropriate catalyst carriers are of great significance to improving the dispersibility of active components. Common catalyst carriers include mesoporous aluminum silicates, graphene, zeolite molecular sieves (ZMS), biomass-activated carbon, and natural minerals [29,30,31,32]. Carbon-based carriers have attracted much attention because of their advantages of developed pore structures, large specific surface area, and low price in recent years [33,34,35]. To prepare carbon-based carriers with a developed pore structure and good dispersibility of active components have become new research focuses [36,37]. In the preliminary study, we used citric acid residue to prepare the porous carbon material. Its specific surface area exceeds 1000 m^2^/g, and it has suitable mesoporous and microporous structures [3]. These results indicate that it is possible to prepare carbon-based catalysts with good dispersibility of active components.

Here, we prepared catalysts of different Ce-Mn ratios through the IWI with the porous carbon material prepared from citric acid residue as the carrier. Their physical and chemical properties were characterized, and their degrading performances for acetone were studied with a catalyst testing device, and the corresponding degradation mechanisms are discussed as well. The research results offer new ideas and approaches for the efficient and comprehensive utilization of by-products in the bio-fermentation industry and for the development of cheap and high degradation performance catalysts for acetone at low temperatures.

## 2. Materials and Methods

### 2.1. Materials

Phosphoric acid (85%) and manganese sulphate (analytical grade) were obtained from Sinopharm Group (Beijing, China). Cerium nitrate (analytical grade) was purchased from Aladdin Reagent Co., Ltd. (Shanghai, China). Citric acid residue (CAR) waste was collected from a citric acid plant in Shandong Province, China. High-purity standard gases of acetone and nitrogen were obtained from Zhaoge Gas Technology Co., Ltd. (Beijing, China). All solutions were prepared with ultrapure water produced by Milli-Q Academic (Boston, MA, USA).

### 2.2. Catalysts Preparation

The activated carbon (AC) carrier was prepared with citric acid residue waste following the method reported in a previous study [3]. The Ce-Mn mixed impregnation solutions were prepared with the solutions of cerium nitrate and manganese sulfate. The mass fraction of Mn in the mixed solution was maintained at 2.0 wt%, and the mass fraction of Ce in the mixed solution was changed at the molar ratios of Ce/Mn 0.0, 0.2, 0.4, 0.6, 0.8, and 1.0.

To prepare catalysts, 10 g AC carrier was first immersed in 100 mL of the Ce-Mn mixed impregnation solution, and ultrasonic treatment lasted for 1 h at room temperature. Then, the samples were dried in a vacuum drying oven at 60 °C for 4 h to remove residue water. After that, the dried sample was heated to 400 °C at a speed of 10 °C/min and maintained for 3 h in a tube furnace. Finally, the samples were cooled to room temperature and sealed at low temperatures. The samples were labelled as Ce_X_-Mn/AC (x indicates the molar ratio of Ce to Mn).

### 2.3. Catalysts Characterization

The N_2_ adsorption–desorption isotherms and the pore size distribution of Ce_X_-Mn/AC were characterized by an automated gas sorption analyzer (Quantachrome, Nova 2000e, Boynton Beach, FL, USA). Powder XRD patterns of the samples were recorded on an XRD diffractometer (Bruker, D8 ADVANCE, Ettlingen, Germany). The data were recorded at a scan rate of 10°/min, and the diffraction peak of the crystalline phase was measured in a scanning range of 2θ = 10–80° and then compared with the diffraction peak of the standard compound reported in the JCPDS data file. The sample surfaces were analyzed using X-ray photoelectron spectroscopy (XPS) (Thermo Fisher Scientific Inc., Waltham, MA, USA). All binding energies of Ce_3d_, Mn_2p_, and O_1s_ were referenced to the neutral C_1s_ peak at 284.6 eV to compensate for surface charging effects. XPS peak 4.1 software (version number: 4.01) was used to fit the XPS spectra peaks, and the full width at half maximum (FWHM) was maintained constant for all components in a particular spectrum. A scanning electron microscope (SEM) (Merlin compact, Zeiss, Oberkochen, Germany) and a transmission electron microscope (TEM) (FEI, Philips Tecnai F20, Hillsboro, OR, USA) were used to study the dispersity of active components on the catalyst sample surfaces.

### 2.4. Catalytic Experiments

**Figure 1:**
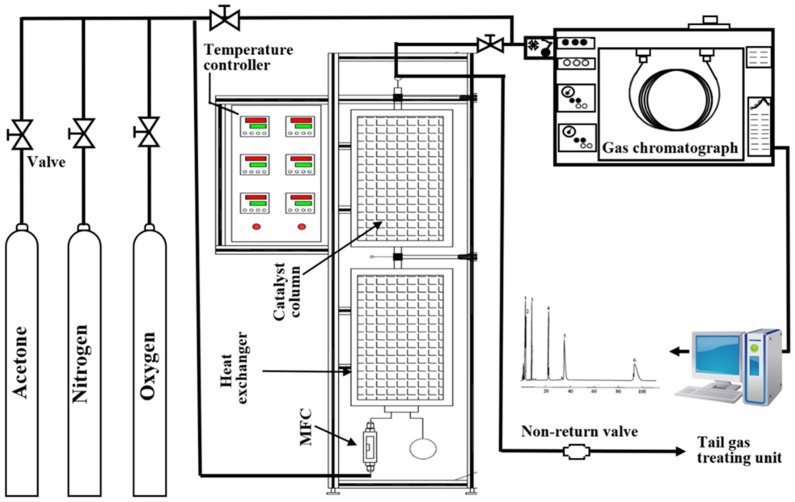
Configuration of the experimental apparatus.

Acetone catalytic degradation experiments were carried out in a fixed-bed catalytic setup, and the experimental apparatus is shown in Figure 1. The prepared high-purity standard gases of acetone were placed in gas cylinders. When the experiment started, the gas channel was opened by a valve, and the mass flow controllers adjusted the flow rate of the stream at 100 mL/min. The gas stream was preheated to 80 °C before entering the catalytic bed. Then, 0.5 g of outgassed catalyst was loaded into a catalytic bed (φ 6 mm × 150 mm). A filter plate was located at the bottom of the column as a support of the catalytic bed. The inlet concentration of acetone (C_in_) was maintained at 238 mg/m^3^ (100 ppm) during the degradation experiments, and the acetone vapor flowed through the fixed bed from the bottom of the catalytic bed. The catalytic reaction temperature was controlled at 25, 80, 120, 170, 220, 250, and 280 °C. The temperature was kept constant for 30 min at each temperature test point in order to determine the degradation conversion of catalysts for acetone. In addition, the optimum catalyst for acetone conversion was selected to study the degradation of acetone under different catalytic reaction temperatures and different oxygen contents in waste gas.

The inlet concentrations (C_in_, mg/m^3^) and outlet concentrations (C_out_, mg/m^3^) of acetone were measured by a gas chromatography instrument (Techcomp GC7900, China) equipped with a TM-624 quartz capillary column (length × inner diameter × thickness, 60 m × 0.32 mm × 2.0 μm) and a flame-ionization detector. Operating conditions were as follows: oven temperature, 65 °C; detector temperature, 220 °C; carrier gas, N_2_ at 20 mL/min. The acetone conversion (ω, %) was calculated as follows [38,39]:

ω = (1 − C_in_/C_out_) × 100% (1)

## 3. Results and Discussion

### 3.1. Catalyst Characterization

**Figure 2:**
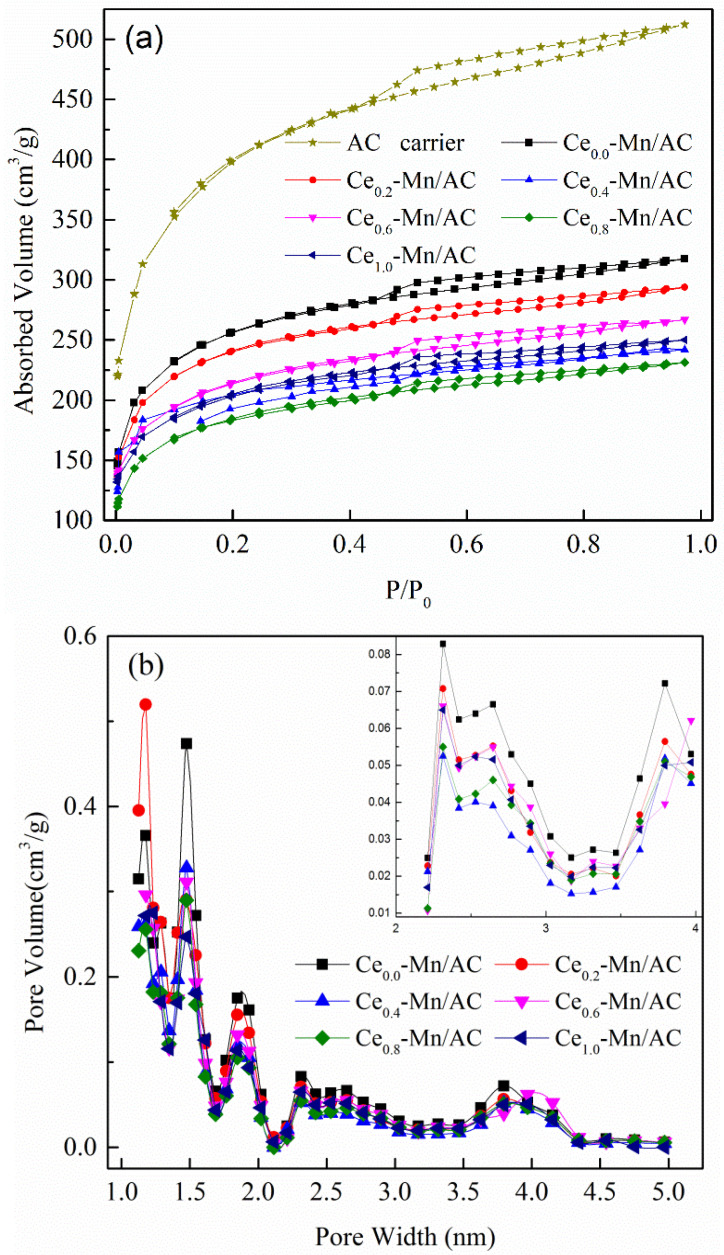
N_2_ adsorption–desorption isotherms (**a**) and DFT pore size distributions (**b**) for samples.

Figure 2a shows the N_2_ adsorption–desorption isotherms of the AC carrier and prepared catalysts. According to the ICPUC classification of adsorption isotherms, the N_2_ adsorption–desorption isotherms of the AC carrier and the prepared carbon-based catalysts are IV-type adsorption isotherms [40]. The hysteresis loops present in the adsorption–desorption isotherm curves between the partial pressures of 0.4 and 1.0 because of the mesoporous structures in the catalysts [41]. Figure 2b shows the pore size distribution of the AC carrier and catalysts. The pore sizes are mostly at 1.2, 1.5, 1.8, 2.3, or 3.8 nm for the AC carrier. After carrying active components, the pore volume of the catalysts is more minor than the original AC carrier because of the deposition of active components onto the AC surfaces [42,43,44]. Table 1 shows the specific surface areas and pore volumes of the prepared catalyst materials. The AC carrier features a large specific surface area and a rich microporous structure. The specific surface area and pore volume of the AC reach 1400.2 m^2^/g and 0.793 cm^3^/g, respectively. The specific surface areas and pore volumes of all catalysts were reduced after carrying active components. Among them, Ce_0.8_-Mn/AC showed maximizing reduction in the specific surface area and pore volume, dropping to 645.18 m^2^/g and 0.358 cm^3^/g, respectively. The results indicate that the active components were successfully loaded on the carriers. Moreover, an appropriate Ce/Mn molar ratio better helps to load the active components onto the carriers [45,46].

**Figure 3:**
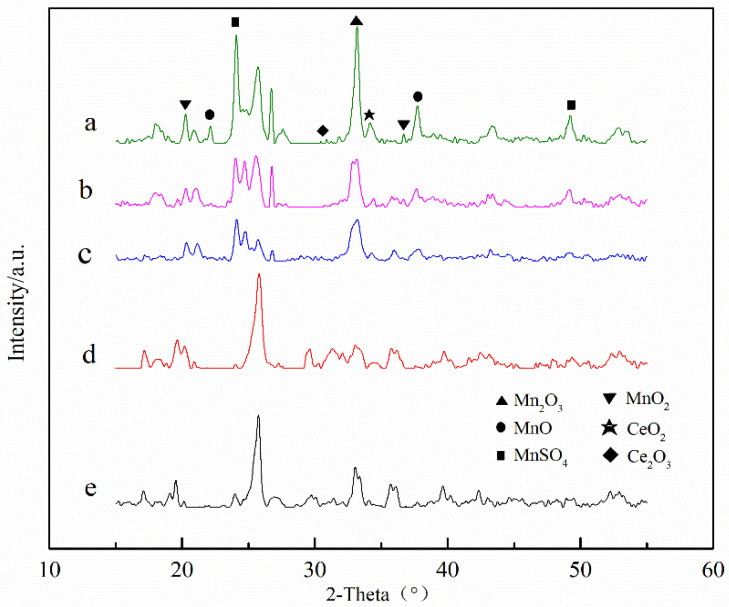
XRD experimental results of different catalysts. (**a**) Ce_0.0_-Mn/AC; (**b**) Ce_0.2_-Mn/AC; (**c**) Ce_0.4_-Mn/AC; (**d**) Ce_0.8_-Mn/AC; (**e**) Ce_1.0_-Mn/AC.

X-ray diffraction (XRD) results of the catalysts are shown in Figure 3. Catalysts of different Ce-Mn ratios were relatively consistent in diffraction peak position and peak pattern. All samples showed sharp characteristic diffraction peaks at 2θ = 24.0° and 49.1°, whose position corresponds to the standard card JCPDS PDF#73-1361, indicating the presence of MnSO_4_ in the samples. All samples showed characteristic diffraction peaks at 2θ = 21.7° and 37.9°, indicating the presence of MnO in the samples (JCPDS 12-0141). In addition, the map of diffraction peaks shows characteristic peaks of Mn_2_O_3_ and MnO_2_ (JCPDS 41.1442, JCPDS 42-1169). These results indicate that manganese was successfully loaded onto the carriers, and part of the MnSO_4_ was converted into MnO_x_ (including Mn_2_O_3_ and MnO_2_) during the firing process. Additionally, after Ce doping, the absorption peaks of CeO_2_ and Ce_2_O_3_ (PDF 34-0394, PDF 44-1086) presented in the diffraction peaks of the catalyst samples [40,47], indicating the conversion of ceric nitrate into CeO_2_ and Ce_2_O_3_. The signal of the MnO_x_ absorption peaks was weakened, indicating that Ce doping can improve MnO_x_ dispersibility in the carriers [47,48,49].

**Figure 4:**
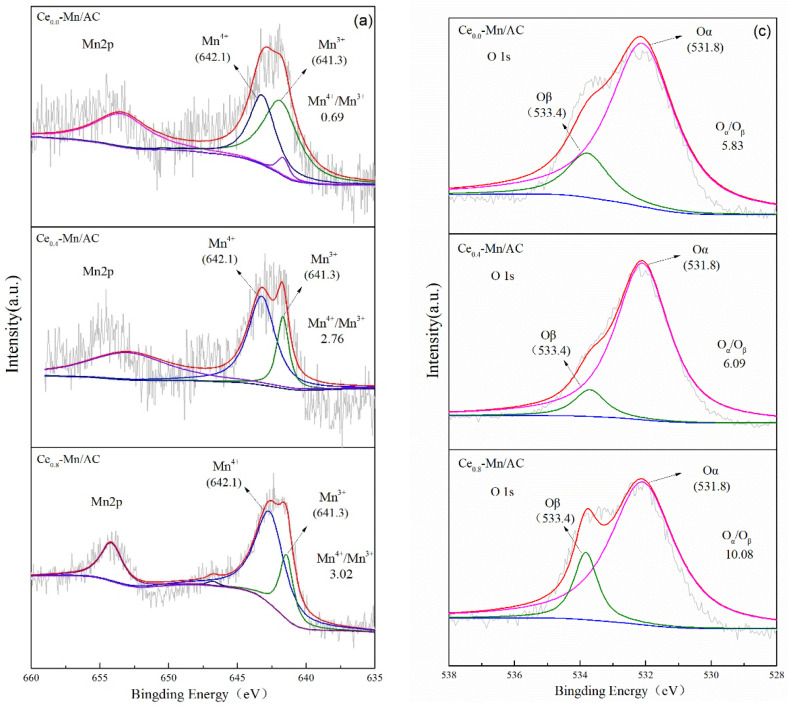

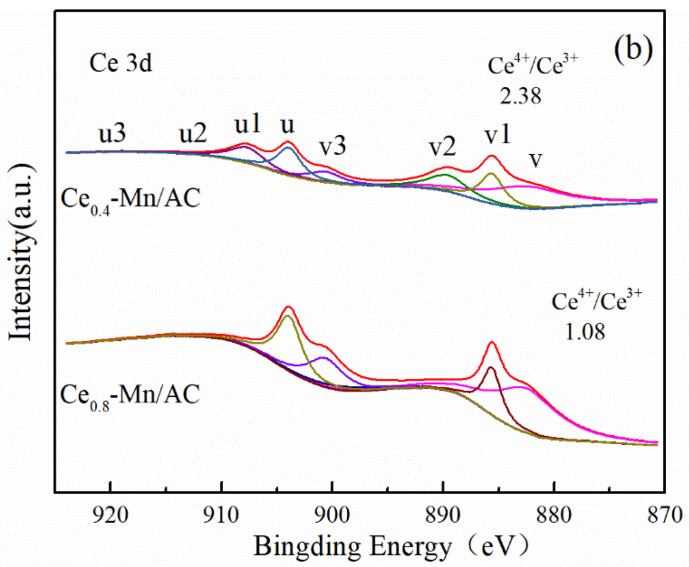
XPS Mn_2p_ (**a**), Ce_3d_ (**b**) and O_1s_ (**c**) spectra on the catalysts.

Figure 4 presents the XPS maps of Mn_2p_, Ce_3d_, and O_1s_ in the Ce_0.0_-Mn/AC, Ce_0.4_-Mn/AC, and Ce_0.8_-Mn/AC samples. For the XPS maps of Mn_2p_ (Figure 4a), the fitted peaks with binding energies of 639.0, 641.3, and 642.4 eV correspond to Mn^2+^, Mn^3+^, and Mn^4+^, respectively [26,50]. The content of oxidizing Mn^4+^ and Mn^3+^ in different samples was calculated by peak fitting software. As the proportion of Ce increased in the sample preparation process, Mn^4+^/Mn^3+^ increased from 0.69 to 3.02, and the content of Mn^4+^ with higher oxidizing performance also increased gradually. A higher proportion of Mn^4+^ is more conducive to the oxidation of organic matter [51]. For the XPS maps of Ce_3d_ (Figure 4b), the Ce_3d_ spectrum can be broken down to eight peaks according to relevant literature [25,36,52,53,54,55], including four pairs of spin-splitting primes of Ce_3d_ that are marked as v and u. Among them, v, v_1_, v_2_, and v_3_ are the spin orbits of Ce_3d_5/2, and u, u_1_, u_2_, and u_3_ are the spin orbits of Ce_3d_3/2. The (u_1_ + v_1_) binding energy peak is attributed to the 3d^10^4f^1^ electronic state of Ce^3+^, and the (u + u_2_ + u_3_ + v + v_2_ + v_3_) binding energy peak is attributed to the 3d^10^4f^0^ electronic state of Ce^4+^. The Ce^3+^ content represents the number of oxygen vacancies on the catalyst surface; the higher content of Ce^3+^, the more oxygen vacancies there are [37,56,57]. Ce^3+^ and Ce^4+^ were found on the surface of both Ce_0.4_-Mn/AC and Ce_0.8_-Mn/AC, and the ratio of Ce^4+^/Ce^3+^ was 2.38 and 1.08, respectively. In addition, Ce^3+^ on Ce_0.8_-Mn/AC was higher than that on Ce_0.4_-Mn/AC [48]. Therefore, higher cerium content is conducive to the formation of more oxygen vacancies on the catalyst surface, and better helps to increase the oxygen transfer rate in the later catalytic degradation of acetone [51,58]. For the XPS maps of O_1s_ (Figure 4c), the fitted peaks with a binding energy of 531.8 and 533.7 eV correspond to lattice oxygen (O_α_) and adsorption oxygen (O_β_), respectively [47,53,59]. As the Ce increased, the numbers of both adsorption oxygen and lattice oxygen on the catalyst surface, as well as the proportion of lattice oxygen, increased. The O_α_/O_β_ ratio on the Ce_0.8_-Mn/AC surface reached 10.08. This is partly due to the fact that a higher content of Mn^4+^ is more attractive lattice oxygen, and a higher content of Ce^3+^ is more likely to form oxygen vacancies [60].

**Figure 5:**
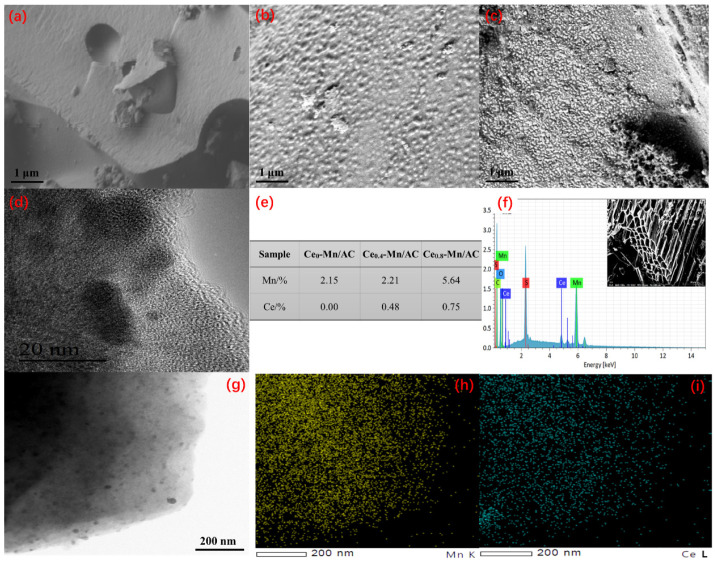
Scanning electron microscope (SEM) images of AC (**a**); Ce_0.4_-Mn/AC (**b**); Ce_0.8_-Mn/AC (**c**); transmission electron microscope (TEM) images of Ce_0.8_-Mn/AC (**d**,**g**); elemental contents of different catalysts (**e**); EDS spectra of Ce_0.8_-Mn/AC (**f**); EDS elemental mapping of Mn (**h**) and Ce (**i**) on the Ce_0.8_-Mn/AC.

SEM and TEM images of AC carrier, Ce_0.4_-Mn/AC, and Ce_0.8_-Mn/AC are shown in Figure 5. The original AC carrier presents a smooth surface (Figure 5a). After adding the active components to the AC carrier surface, the sample surface showed many highly dispersed raised structures. Samples of Ce_0.8_-Mn/AC (Figure 5c) showed significantly more raised structures than Ce_0.4_-Mn/AC (Figure 5b). The EDS results (Figure 5e,f) show that the Mn and Ce content on the surface of Ce_0.8_-Mn/AC samples reached 5.64% and 0.75%, respectively, obviously higher than those of the Ce_0.4_-Mn/AC samples. TEM test results of Ce_0.8_-Mn/AC samples are shown in Figure 5d,g. A large amount of particulate metal oxide with different sizes was found and was highly dispersed on the surface of the AC. That larger ones with a diameter of about 10 nm interlace to the small ones with a diameter of 2–3 nm. According to the EDS element mapping of Mn (Figure 5h) and Ce (Figure 5i), all catalyst surfaces showed a large amount of Mn and Ce particles, which were highly and uniformly dispersed. This indicates that adding Ce helps improve Mn dispersibility on the surface of catalyst carriers, and highly dispersed Mn and Ce particles can further provide more active sites for the subsequent catalysis and enhance the overall catalytic performance [61,62].

### 3.2. Catalytic Performance of Catalysts for Acetone

**Figure 6:**
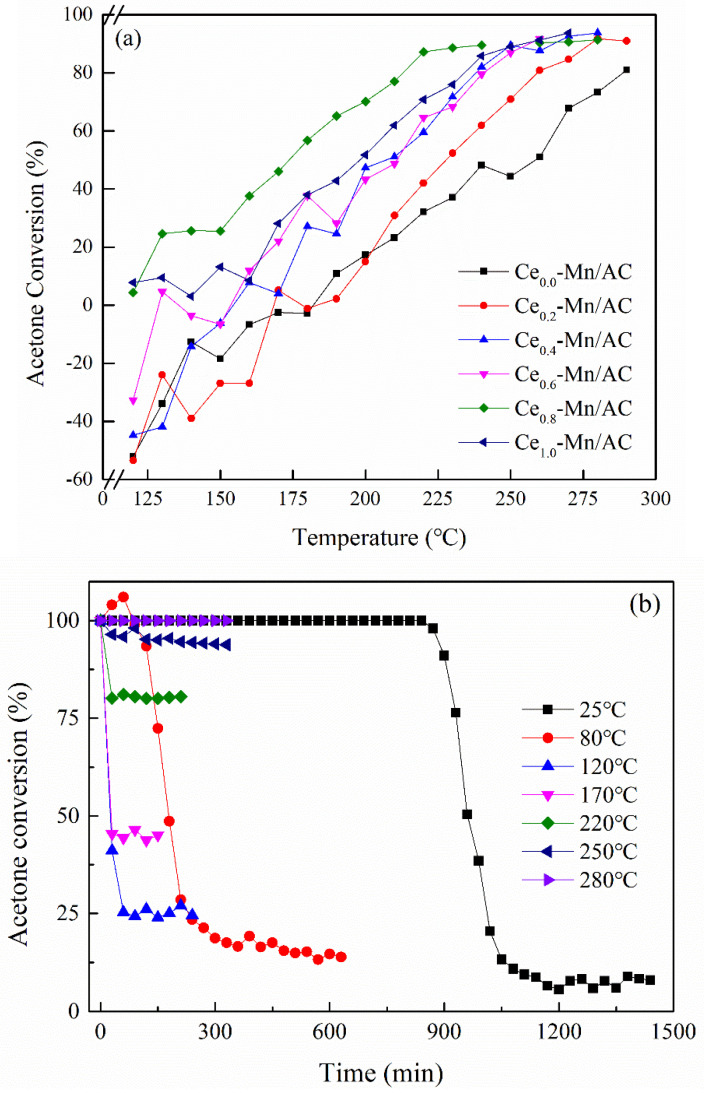

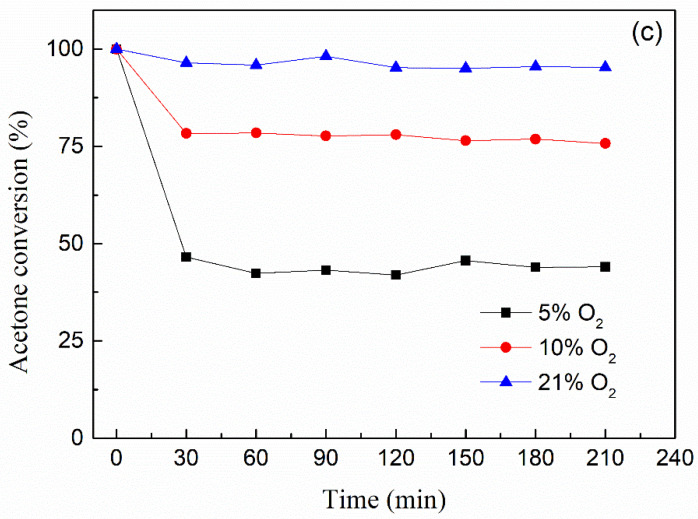
The acetone conversion of the catalysts for acetone (**a**); effects of reaction temperature on the removal efficiency of acetone over Ce_0.8_-Mn/AC as a function of time (reaction conditions: acetone concentration = 238 mg/m^3^, oxygen content = 21%) (**b**); effects of oxygen content on the removal efficiency of acetone over Ce_0.8_-Mn/AC as a function of time (reaction conditions: acetone concentration = 238 mg/m^3^, reaction temperature = 250 °C) (**c**).

At the acetone concentration of 238 mg/m^3^ and the acetone conversion in the catalysts at different temperatures are shown in Figure 6a. When the temperature was below 175 °C, the acetone conversion of some samples was beneath zero, which is attributed to the adsorbed acetone in the porous structure at low temperatures released with the temperature increase, thus leading the outlet concentrations of acetone to be higher than the inlet concentrations. Taking the corresponding temperature at the acetone conversion of 50% (T_50_) and that at the acetone conversion of 90% (T_90_) as the criteria for catalyst activity, the catalyst activity of these samples is in the proper order of Ce_0.8_-Mn/AC, Ce_0.4_-Mn/AC, Ce_0.6_-Mn/AC, Ce_0.2_-Mn/AC, Ce_1.0_-Mn/AC, and Ce_0.0_-Mn/AC. Ce_0.8_-Mn/AC showed the best acetone catalytic activity, and T_50_ and T_90_ were 175 and 245 °C, respectively. This is related to the highest dispersity of Mn and Ce particles on Ce_0.8_-Mn/AC surfaces. Comparison with the acetone conversion of a similar types of catalysts reported by Lin and Liang-Yi [63] and Rezlescu [28] indicates that the Ce_0.8_-Mn/AC prepared in this study exhibits excellent catalytic activity for acetone. Figure 6b shows the acetone degradation rate of Ce_0.8_-Mn/AC over time under different catalyzing temperatures. At room temperature (25 °C), the acetone conversion was first maintained at 100%. After 900 min, it declined gradually over time and finally maintained at a low level. This is mainly due to the acetone adsorption at a low temperature. As the temperature increases, the breakthrough time and amount of adsorption of acetone decrease because the acetone adsorption in carrier is an exothermic process [64]. At the same time, the catalytic activity of Ce_0.8_-Mn/AC for acetone increases. When the temperature exceeds 250 °C, the acetone conversion of Ce_0.8_-Mn/AC surpasses 90%, and at 280 °C, the acetone conversion is nearly 100%. At 250 °C, the conversion of Ce_0.8_-Mn/AC for acetone over time at different oxygen contents is shown in Figure 6c. As the oxygen content decreased, the acetone conversion was reduced as well. Lower oxygen content in the waste gas leads to fewer lattice oxygen and adsorption oxygen around Mn^4+^ and Ce^3+^ [65], and thus decreases the oxygen transfer rate in acetone degradation.

### 3.3. Mechanism of Catalytic Oxidation of the Ce_0.8_-Mn/AC

In recent years, researchers have delved into the catalytic oxidation mechanism of VOCs [66]. Active component particles coat the carrier surface as support, and VOC reactants are adsorbed onto the material surface. Adsorbed VOCs first react with lattice oxygen or adsorption oxygen on the surface of the catalyst carrier to form active intermediates. Then, the intermediaries desorb from the catalyst surfaces and react with each other to form inorganic compounds. Meanwhile, active sites losing lattice oxygen or adsorption oxygen can capture gaseous oxygen in the air to form lattice oxygen and adsorption oxygen again, and thereby regenerate the catalyst.

**Figure 7:**
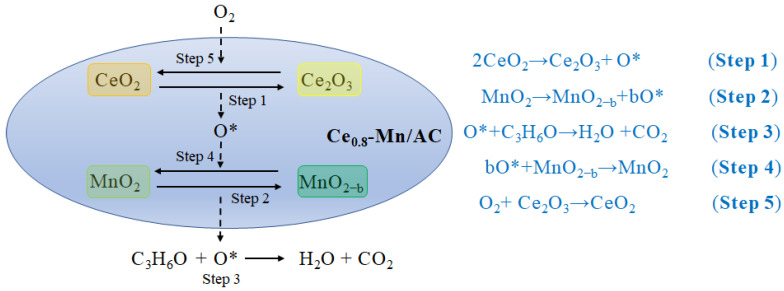
Schematic of catalysts of the acetone on Ce_0.8_-Mn/AC.

The reaction mechanism is shown in Figure 7. Highly dispersed CeO_2_ on the catalyst surface provides large amounts of oxygen vacancies in the catalytic process. Ce atoms can connect with Mn and O and form the Mn-O-Ce bridge, and further reinforce Mn-O_2_ binding. The bridge serves as a channel for the electron transfer between manganese and cerium cations [45,67]. During the thermal decomposition of CeO_2_ into Ce_2_O_3_ (Step1) [61], a large amount of adsorption oxygen is released, and thus it accelerates the conversion of Mn^3+^ into Mn^4+^ (Step4) [45,67]. In the meantime, it ensures timely oxygen atom supply to the surface when most of the lattice oxygen on the Mn^3+^/Mn^4+^ surface is consumed [60]. Mn^3+^ and Mn^4+^ exhibit high oxidizing ability, and can release active oxygen (O*) when reacting with acetone (Step2). Active oxygens react with acetone to form final degradation products (Step3) [66]. In addition, oxygen atoms in the air promptly make up the oxygen vacancies formed after reacting on the catalyst surface, and finish catalyst regeneration (Step5) [31,61,68]. The study results indicate that cerium oxide and manganese oxide are synergistic in the catalytic degradation of acetone. Adding cerium to manganese-based catalysts can increase the oxygen migration rate and lattice oxygen reduction rate.

## 4. Conclusions

With a porous carbon material prepared from citric acid residue as the carrier, the catalysts of different Ce-Mn ratios were prepared using the IWI method. When the molar ratio of Ce/Mn was 0.8, a large amount of Mn and Ce particles were highly and evenly distributed on the prepared catalyst Ce_0.8_-Mn/AC surfaces. The Mn and Ce contents on the Ce_0.8_-Mn/AC surface reached 5.64% and 0.75%, respectively. At the acetone concentration of 238 mg/m^3^, the catalytic activity of Ce_0.8_-Mn/AC for acetone was the best, and T_50_ and T_90_ values were 175 and 245 °C, respectively. During the degradation process, adding cerium to manganese-based catalysts improves the oxygen migration rate and the lattice oxygen reduction rate. The synergistic action of Ce and Mn improves the catalytic performance in acetone degradation at low temperatures. The research results provide new ideas for developing carbon-based catalysts for the efficient degradation of acetone.

## Figures and Tables

**Table 1:** Porous structural parameters of samples.

Catalyst	S_BET_ (m^2^/g)	Mesoporous Volume (cm^3^/g)	Total Pore Volume (cm^3^/g)
Biomass-activated carbon	1400.25	0.179	0.793
Ce_0.0_-Mn/AC	918.07	0.096	0.491
Ce_0.2_-Mn/AC	837.83	0.084	0.454
Ce_0.4_-Mn/AC	702.42	0.071	0.376
Ce_0.6_-Mn/AC	776.49	0.084	0.413
Ce_0.8_-Mn/AC	645.18	0.074	0.358
Ce_1.0_-Mn/AC	725.98	0.113	0.387
